# Treatment of cold intolerance following finger pulp amputations: a case comparison between immediate finger replantation and delayed pulp and digital arterial arch reconstruction with flow-through free hypothenar flap

**DOI:** 10.1080/23320885.2021.2020656

**Published:** 2021-12-27

**Authors:** Ryohei Ishiura, Makoto Shiraishi, Yoshimoto Okada, Kohei Mitsui, Chihena Hansini Banda, Kanako Danno, Mitsunaga Narushima

**Affiliations:** Department of Plastic and Reconstructive Surgery, Mie University, Tsu, Japan

**Keywords:** Digital amputation, digital replantation, hypothenar flap, digital arterial arch, cold intolerance

## Abstract

We report a unique case of cold intolerance following identical fingertip amputations of two fingers on the same hand. The index finger was replanted and the middle finger was reconstructed with a free flow-through hypothenar perforator flap to anatomically restore the digital arterial arch circulation and successfully treat cold intolerance.

## Introduction

Cold intolerance is one of the most common intermediates to long-term complications of injury, reconstruction and replantation of fingers. It is often accompanied by other symptoms such as pain, skin color change, numbness, swelling, weakness, stiffness, and loss of finger function. At present, the pathophysiology remains poorly understood with the most prominent theory being a disorder in extremity blood circulation [1–3]. However, inconsistency in the development of cold intolerance across the various types of finger injuries and reconstruction techniques has resulted in disputes on the optimal treatment approach.

We report a unique case of cold intolerance following identical fingertip amputations of two fingers on the same hand-treated using different approaches. The index finger was replanted and the middle finger was reconstructed with a free flow-through hypothenar perforator flap to anatomically restore the digital arterial arch circulation and successfully treat cold intolerance.

## Case report

**Figure 1.**
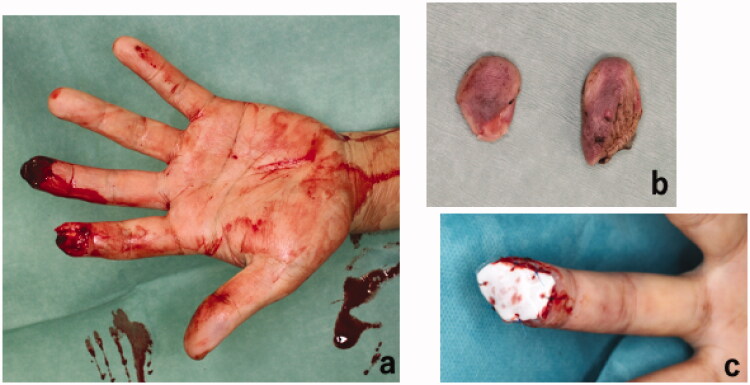
Clinical photograph of the hand following injury showing (a) identical volar oblique amputations of the left index and middle fingers. (b) Shows the amputated segments of the left index and middle fingertips. (c) Debridement and coverage of the middle finger stamp with artificial dermis after unsuccessful replantation. Index finger replantation was successful.

We report a 51-year-old non-smoking man who presented with left index and middle fingertip amputations. He sustained the injuries while falling from a height when his fingers became entrapped between blocks of a wall in an effort to reach for support. This resulted in volar oblique avulsion of his finger pulps distal to the distal interphalangeal joint crease (Figure 1). There were no fractures and the distal phalanxes were intact. On the day of injury, we performed emergency replantation surgery for both fingers under digital nerve block. A single artery and vein were anastomosed for each fingertip. Index finger replantation was successful, but the middle fingertip underwent necrosis. The patient declined further reconstructive surgery of the middle finger stump. Therefore, debridement and coverage with artificial dermis (Terudermis, Alcare, Japan) were performed instead and the wound healed by secondary intention (Figure 1).

Following discharge, cold intolerance developed in both fingers resulting in discomfort and difficulty in performing his job, particularly when typing. 1 year after the initial injury, he returned for middle finger reconstruction to restore finger length and treat cold intolerance but was unwilling to have any further surgery on the replanted index finger. To reconstruct the finger pulp with the anatomical restoration of blood circulation, we performed a free flow-through hypothenar perforator flap for the reconstruction of the left middle finger pulp and the digital arterial arch.

**Figure 2.**
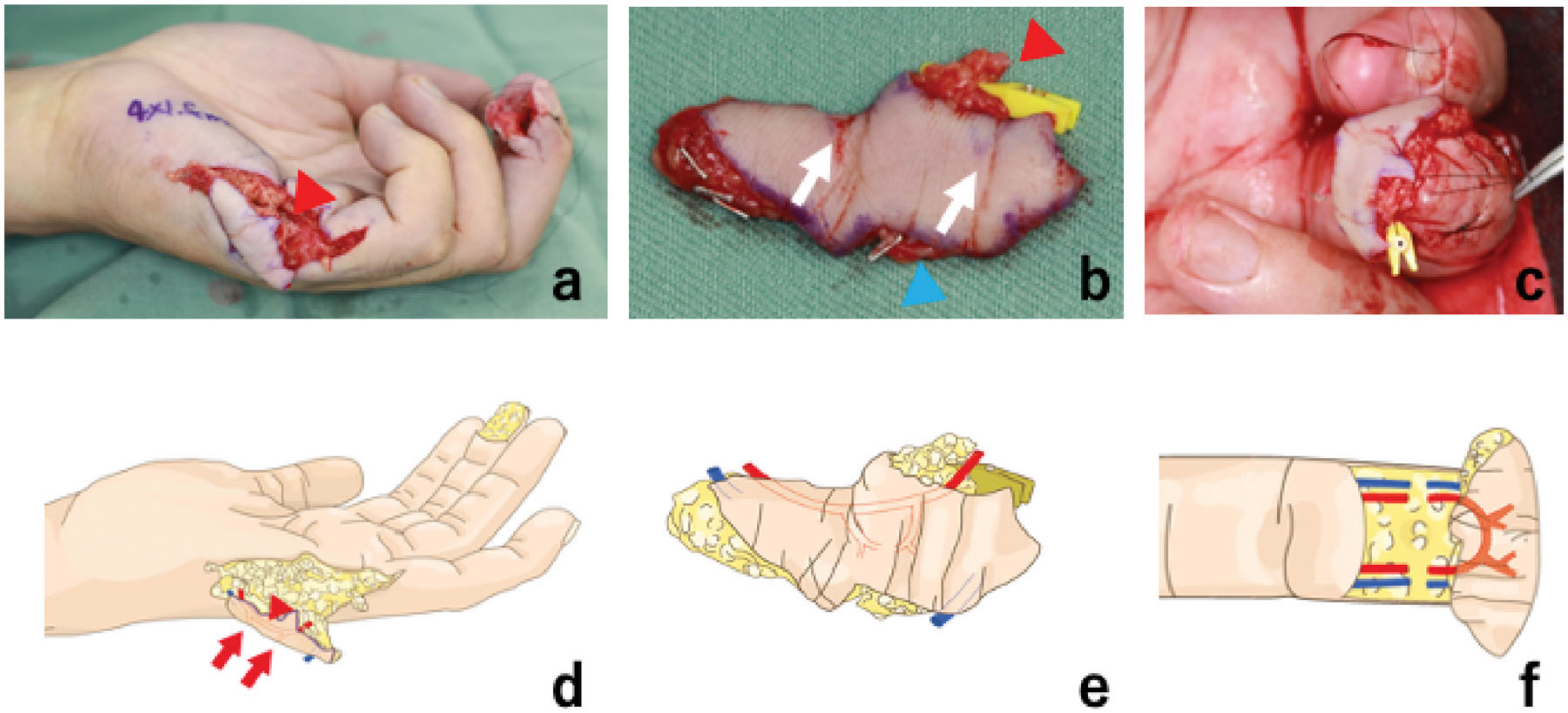
Intraoperative photographs and illustrations showing (a) and (d) the 40 × 15 mm free flow-through hypothenar perforator flap design and elevation on the ipsilateral hand. The red arrowhead shows the pedicle artery. The elevated free flap (b) and (d) showing the pedicle artery (red arrowhead), the skin perforators (white arrows), and one of the two cutaneous veins for venous drainage (blue arrowhead). Insertion of the harvested free flap (c) on the left middle fingertip. Reconstruction of the digital arterial arch using the flow-through hypothenar flap (f). The pedicle vessel was anastomosed in flow-through anastomoses fashion to the bilateral digital arteries. The cutaneous veins were anastomosed to the bilateral digital veins in end-to-end fashion.

Preoperatively, locations of the hypothenar perforators were identified by color doppler ultrasound (APLIO 500 TUS − A500, Toshiba, Japan) and marked. Reconstruction was performed under general anesthesia and hemorrhage controlled with a pneumatic tourniquet. A 40 × 15 mm hypothenar perforator flap was designed in the ipsilateral hand and incised from the ulnar side. The flap was elevated above the palmaris brevis fascia with two artery perforators and two cutaneous veins included in the flap. The perforators were then dissected proximally and the pedicles prepared. The diameter of the artery pedicle was 1 mm on the proximal side and 1 mm on the distal side. A pedicle length of 15 mm was included in the flap for reconstruction of the digital arterial arch. The middle finger was prepared for the transfer with bilateral mid-lateral incisions and excision of the scar tissue. Bilateral proper palmar digital arteries and palmar digital veins were prepared for the anastomoses. The diameters of the recipient arteries were 1 mm on the ulnar side and 0.9 mm on the radial side. The diameters of the recipient’s veins were 1.2 mm on the ulnar side and 1 mm on the radial side. The harvested flap was placed on the prepared site with the distal side of the flap on the ulnar side of the fingertip. Both arteries were anastomosed in end-to-end fashion in flow-through style and both veins were also anastomosed end-to-end in antegrade fashion (Figure 2). The donor site was closed primarily. Intravenous Prostaglandin E_1_ analog (Apistandin, Fuji Pharma Co., Ltd, Tokyo, Japan) was administered at 40 micrograms a day for a week.

**Figure 3.**
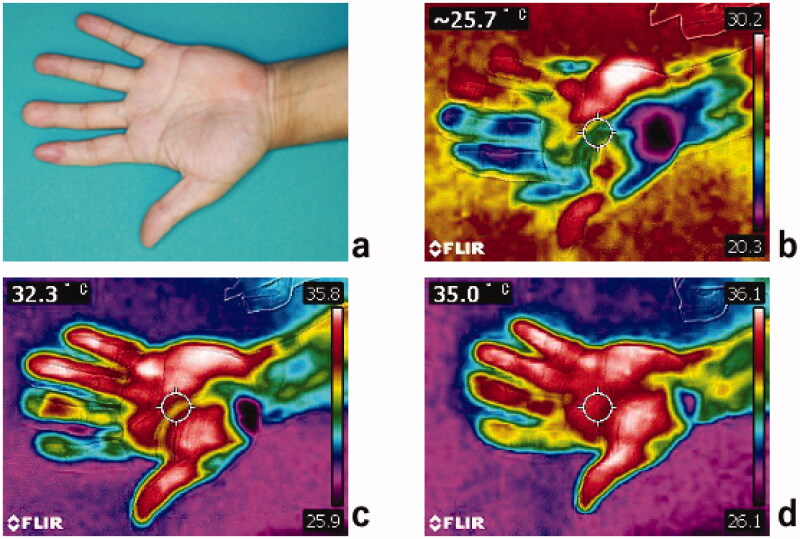
Clinical photography of the patient’s left hand at 3 years follow-up showing (a) the replanted index finger and the middle finger reconstructed using a free flow-through hypothenar flap. Cold stress test thermography; (b) immediately after immersion in cold water, (c) 1 min post immersion and (d) 2 min post immersion.

The postoperative course was uneventful, and the flap survived completely with no complications. Debulking surgery was performed under local anesthesia 28 months after the flap transfer. Follow-up at 3 years showed exceptional hand cosmesis with good flap contour and skin color, durability and texture matching. Semmes–Weinstein (SW) monofilament test results of the replanted index finger, the reconstructed middle finger and the donor site were 2.44, 2.83 and 2.83 respectively. Cold intolerance completely resolved in the middle finger but some discomfort persisted in the index finger. A cold stress test was then performed to assess hand sensitivity as described by Harada et al. [4]. Briefly, following rest for 20 min in the examination room (23–25 °C), the hand was immersed into cold water (10 °C) for 10 min. The temperature of the hand was assessed using an infrared thermometer (FRIR C2, Teledyne FLIR LLC, United States) immediately after immersion, at 1 min and 2 min post immersion. The reconstructed middle finger showed faster recovery than the replanted index finger (Figure 3). The patient returned to work satisfied with the result and reported improved hand function.

## Discussion

The reported incidence of cold intolerance following digital amputation with or without replantation remains high with more than 80% of patients developing symptoms [5–7]. A recent retrospective study of fingertip injuries by van den Berg et al. comparing results of reconstruction, bone-shortening, and conservative therapy revealed no difference in its occurrence rate across these treatment methods [7]. These results highlight the longstanding challenges in understanding the etiology, prevention and treatment of cold intolerance. Notably, these studies were limited by inherent bias in patient allocation to the various treatment methods based on the severity of the injury. This case report of two identical amputations in the same hand presents a unique comparison of long-term development and treatment of cold intolerance in replanted and flow-through flap reconstructed fingers.

The most widely accepted theory of the mechanism of cold intolerance attributes disorders in extremity circulation as the underlying cause. In support of this theory, Nylander et al. investigated finger systolic pressure in finger amputation patients with cold intolerance and found changes in finger vasoregulation were associated with symptoms [2]. Similarly, Gang et al. reported decreased digital blood flow, including volumetric flow rate and skin blood flow observed in finger replanted patients with cold intolerance [1]. Tark et al. reported replanted fingers with a single artery anastomosis suffered more from cold intolerance than those who had two artery anastomoses [3]. Together, these studies demonstrate that both blood volume flow and physiologic local vasoregulation are important in the development of cold intolerance. In our case, the index finger was replanted with a single artery and vein anastomosis due to the limited duration of the digital block anesthesia. Based on these findings, we hypothesized that a more anatomical correction of distal finger pulp circulation including reconstruction of the digital arterial arch may decrease the risk of cold intolerance. As a result, we designed a flow-through hypothenar flap as the optimal flap to achieve this goal and treat cold intolerance in the middle finger.

Various techniques for finger pulp reconstruction have been utilized worldwide. Traditionally, V-Y advancement flaps, cross-finger flaps and thenar flaps are considered the gold standard while the advancement of microsurgery techniques has resulted in increased utilization of partial toe-to-hand transfers and digital artery perforator (DAP) flaps. Recently, the free hypothenar perforator flap has emerged as a useful alternative with many advantages in the reconstruction of finger pulp over other techniques [8]. Using this flap, the complete reconstruction procedure can be performed in one stage on a single operative field. The flap provides identical glabrous skin matching that of the fingertip and the potential flap size is significantly larger than DAP flaps [9] with Seo et al. reporting flap harvest of up to 7 × 2 cm of skin [8]. Cadaver dissection studies have demonstrated that one pedicle has on average seven skin perforators [10]. We used this anatomical feature in our patient to design a novel flow-through hypothenar flap to reconstruct the digital arterial arch across the bilateral digital arteries while also achieving increased finger length and exceptional aesthetic results. The complete resolution of the symptoms associated with cold intolerance as well as the faster cold stress test recovery of the flow-through flap reconstructed finger compared to the replanted finger, in this case, support the circulation theory in the development of cold intolerance. However, the recovery of sensation at 3 years was slightly better in the replanted finger (SW = 2.44) than the flap reconstructed finger (SW = 2.83).

In conclusion restoration of a physiological fingertip circulation should be considered in the treatment of cold intolerance following fingertip amputations. The free flow-through hypothenar perforator flap is a useful option for digital arterial arch reconstruction, treatment of cold intolerance and fingertip reconstruction. Further studies with more cases are required to confirm the efficacy and clinical application of this technique.
